# A rapid liquid chromatography-tandem mass spectrometry based method for the detection of Tet(X) resistance gene in *Enterobacteriaceae*

**DOI:** 10.3389/fmicb.2024.1477740

**Published:** 2024-12-05

**Authors:** Liyun Zhang, Jie Xie, Ziyu Qu, Duan Duan, Chujun Liu, Di Zhang, Haiyang Jiang, Xinhua Dai, You Jiang, Xiang Fang, Congming Wu

**Affiliations:** ^1^National Key Laboratory of Veterinary Public Health and Safety, College of Veterinary Medicine, China Agricultural University, Beijing, China; ^2^Technology Innovation Center of Mass Spectrometry for State Market Regulation, Center for Advanced Measurement Science, National Institute of Metrology, Beijing, China

**Keywords:** tigecycline, tet(X), *Enterobacteriaceae*, LC-MS/MS, detection

## Abstract

There is a major public health threat posed by antibiotic resistance around the world. Tigecycline overcomes the resistance mechanisms of traditional tetracyclines and is often seen as the final resort in combating infections caused by bacteria resistant to multiple drugs. However, the introduction of new mobile tet(X) tetracycline destructases is leading to a notable rise in tigecycline resistance. Therefore, a rapid detection method is needed to monitor the spread of tigecycline resistance. In this study, a novel liquid chromatography-tandem mass spectrometry (LC-MS/MS) method to detect tet(X) in bacterial isolates was developed. This method utilized the analysis by LC-MS/MS of metabolite ratios to determine the presence of tet(X). Bacterial suspensions were co-incubated with tigecycline for 1 h, where tet(X) destructase inactivated tigecycline, making a particular metabolite with a 16-Da change in mass. The characterized quantitative ion pairing of tigecycline in the ESI positive mode was observed at 586.1 → 569.1 m/z. The oxygenated tigecycline detection was established at 602.2 → 529.1 m/z. A model was established using 35 tet(X)-positive and 15 tet(X)-negative *Enterobacteriaceae* strains in this study to optimize the cutoff value. Applying the model to analyze 70 bacterial isolates, the sensitivity of the LC-MS/MS test was 98.9% compared to polymerase chain reaction (PCR), and specificity was 100%. This method is rapid and easy to operate, providing results within 1 h, making it more suitable for routine use in clinical microbiology laboratories.

## 1 Introduction

Tigecycline is considered the first glycylcycline to be marketed. As a representative drug of tetracycline antibiotics, infections that are resistant to multiple drugs are regularly treated with this drug (Tasina et al., 2011; Yaghoubi et al., 2022). The resistance mechanism of tigecycline is different from other tetracycline antibiotics due to its unique structure. Therefore, it is considered the last effective option in clinical practice for treating infections induced by *Enterobacteriaceae* exhibiting resistance to carbapenem. Currently, the global problem is the emergence and a threat of spread of resistant strains with the use of tigecycline, and the resistance mechanisms have become diverse and complex (He et al., 2019; Zha et al., 2020; Jin et al., 2021).

**Figure 1:**
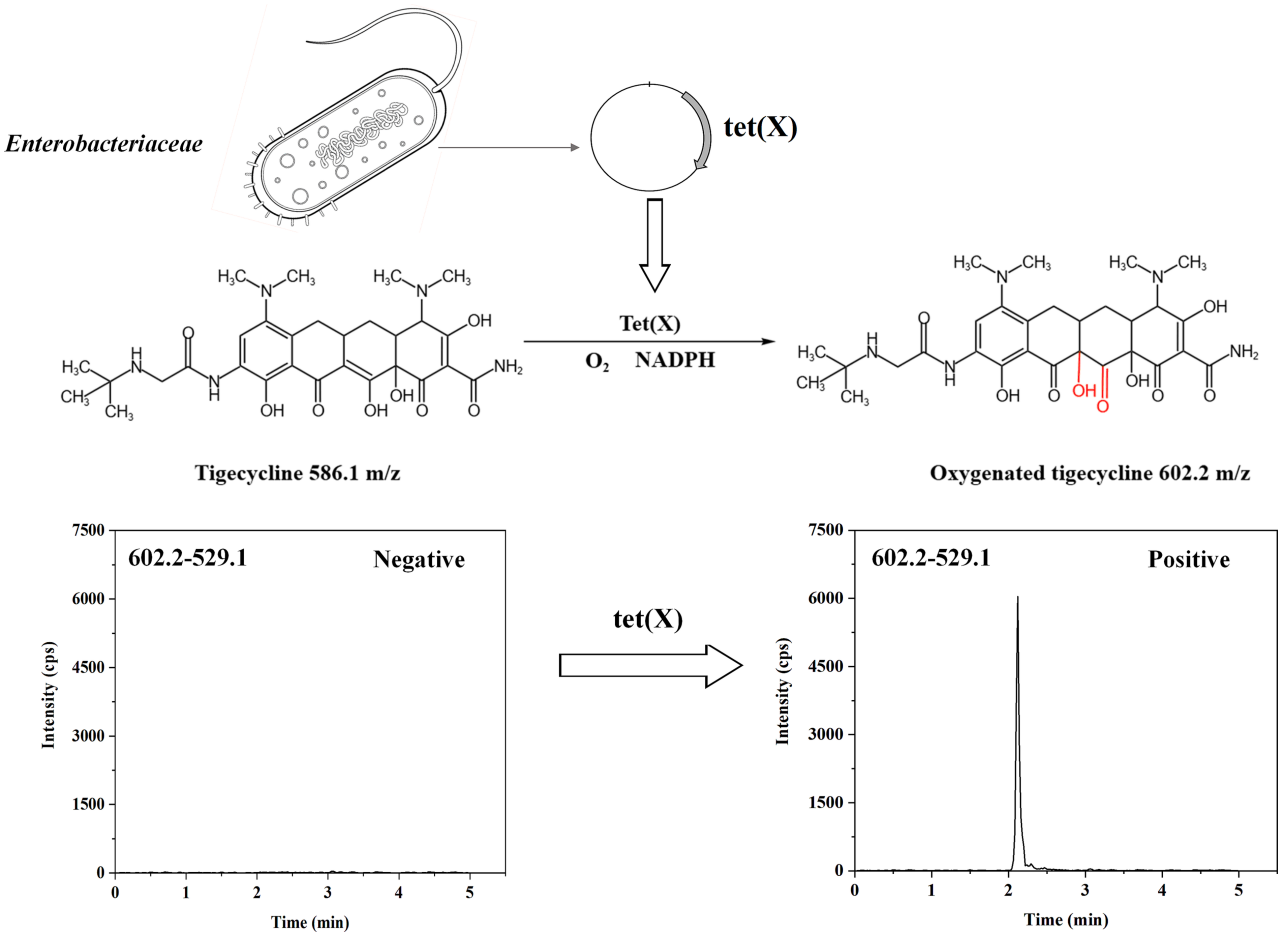
The mechanism by which tet(X) renders tigecycline ineffective.

Given the unique resistance mechanism of tigecycline, delving into the specific causes of its resistance is crucial for formulating effective anti-infection strategies. Tigecycline resistance is primarily found in multidrug-resistant *Enterobacteriaceae* and *Acinetobacter baumannii* bacteria. The overexpression of non-specific efflux pumps encoded by chromosomes is the most common resistance mechanism, which affects the interaction between antibiotics and their targets, and has no impact on tigecycline’s concentration or activity (Doi, 2019; Cui et al., 2020). Conversely, tet(X) are flavin-dependent monooxygenases that first discovered in *Bacteroides fragilis*, The Tet enzyme facilitates the modification of tigecycline (X) by catalyzing a covalent alteration at the C11a site within its tetracycline core structure, which is accompanied by the insertion of an oxygen atom at that location. The resultant product exhibits a mass-to-charge ratio (m/z) of 602 ± 0.2, an increase from the original m/z of 586 ± 0.2 for tigecycline, thereby indicating the successful incorporation of an oxygen atom (Speer and Salyers, 1988). The specific mechanism of action is illustrated in Figure 1. Tet(X3), tet(X4), and tet(X5) have been, respectively, detected in *Acinetobacter baumannii* and *Escherichia coli* since 2019 (Sun et al., 2019; Wang et al., 2019). Bacterias carrying tet(X1) and tet(X2) are resistant to early tetracyclines such as tetracycline, doxycycline, and minocycline, while it has been found that bacterias carrying tet(X3) and tet(X4) are highly resistant to tigecycline. A study cloned the full-length sequences of 7 tet(X) genes into an *Escherichia coli* expression system and evaluated the tigecycline resistance levels of the corresponding *Escherichia coli* strains, showing that *Escherichia coli* harboring tet(X3) and tet(X4) exhibited the greatest levels of resistance (Cui et al., 2021). Moreover, the study showed that the novel mobile tigecycline inactivating enzymes tet(X3) and tet(X4) located on plasmids could deactivate all tetracyclines (He et al., 2019). The entire family of tetracycline antibiotics is at risk because of this situation (Fang et al., 2020). To monitor the dissemination of resistance to tigecycline, a fast and accurate tet(X) detection method is imperative.

Currently, the methods used to detect drug-resistant genes can be mainly divided into two categories. The first category is genotype methods, such as PCR technology and real-time fluorescent quantitative PCR (qPCR) technology (Moore et al., 2005). The second category is phenotype methods, which include MALDI-TOF-MS and LC-MS/MS methods (Li et al., 2015). However, both of these methods have their limitations. Although the detection method based on multiplex real-time PCR is considered the gold standard for detecting drug resistance genes (Fu et al., 2020). PCR technology is limited in its capacity for high-throughput detection, as it requires individual reactions for each target gene. Additionally, PCR may be limited by the availability and specificity of primers used to amplify the target gene (Ali et al., 2018). RT-PCR (real-time PCR), on the other hand, is restricted by the design of primers that specifically target the gene of interest. If suitable primers are unavailable, it may hinders the detection or quantification of the target gene (Forero et al., 2019). A method using MALDI-TOF MS was developed to detect high-level tigecycline-resistant bacteria. However, in this method, the tigecycline incubation concentration was very high, and the incubation time was relatively long (Cui et al., 2020). Compared to MALDI-TOF MS, LC-MS-MS is used at a lower concentration due to its ability to achieve high-precision determination. In recent years, quadrupole-linear ion trap (Q-LIT) tandem mass spectrometry has been widely applied in clinical diagnosis and treatment due to its capability to achieve accurate and efficient measurement of small molecular compounds (Fang et al., 2022). In this study, we used LC-MS-MS to rapidly detect common *Enterobacteriaceae* bacteria producing tet(X) in a lower tigecycline incubation concentration. There is no use of LC-MS-MS to detect the drug resistance gene tet(X).

## 2 Materials and methods

### 2.1 Bacterial strains

A total of 123 strains utilized in this investigation are maintained in our laboratory (Sun et al., 2020). These strains included 93 *Enterobacteriaceae* strains producing tet(X) and 30 non-tet(X) producers. These strains were segregated into three distinct cohorts, with one specially dedicated to establishing thresholds, as denoted in Table 1, constituting a total of 50 strains. Analysis via polymerase chain reaction (PCR) techniques disclosed that 35 bacterial isolates exhibited positivity for the tet(X) gene, while 15 isolates were deemed negative for the same gene. Among the cohort of tet(X) producers, a breakdown revealed the presence of 26 tet(X4) *Escherichia coli* (*E. coli*) strains, 3 tet(X3) *E. coli* strains, and 6 tet(X4) *Klebsiella pneumonia* (*K. pneumonia*) strains. The 15 non-tet(X) producer strains encompassed 14 non-tet(X) *E. coli* strains and 1 non-tet(X) *K. pneumonia* strain (Supplementary Figures S1, S2). Furthermore, another group encompassed a control strain of *E. coli* (DH5α-1-tet(X3)), a *K. pneumonia* control strain (K12106-tet(X4)), and the standard American Type Culture Collection strain 25,922. Validation of the model’s effectiveness was pursued through another subset of strains, detailed in Table 2, comprising a total of 70 strains. PCR analyses unveiled that 56 bacterial isolates displayed tet(X) positivity, contrasting with the 14 isolates that exhibited tet(X) negativity (Supplementary Figures S3, S4). These test strains were collected in the farms and slaughterhouses of pigs and chicken in different regions. All the tested strains were identified using LC-MS-MS.

**Table 1:** Information of establishing model strains.

Species	PCR	QLIT-6610MD metabolic ratio	AB6500+ metabolic ratio	Total number of isolates
*E. coli*	Non-tet(X)	0.002–0.032	0.016–0.061	14
	tet(X4)	0.521–0.879	0.564–0.899	26
	tet(X3)	0.726–0.874	0.737–0.896	3
*K. pneumonia*	Non-tet(X)	0.029	0.033	1
	tet(X4)	0.568–0.781	0.560–0.815	6
	tet(X3)	/	/	/

**Table 2:** Information of the strains used for test validation.

Species	Total number of isolates	PCR	QLIT-6610MD metabolic ratio	AB 6500+ metabolic ratio
Test strains	70			
Tet(X) producers	56			
*E. coli*	49	tet(X4)	0.418–0.908	0.453–0.886
*E. coli*	4	tet(X3)	0.638–0.909	0.704–0.911
*E. coli*	1	tet(X)	0.02	0.025
*E. cloacae*	2	tet(X4)	0.589–0.637	0.630–0.638
Non-Tet(X) producers	14			
*E. coli*	4	non-tet(X)	0.023–0.039	0.021–0.040
*K. pneumonia*	10	non-tet(X)	0.016–0.059	0.011–0.051

### 2.2 Sample pretreatment

**Figure 2:**
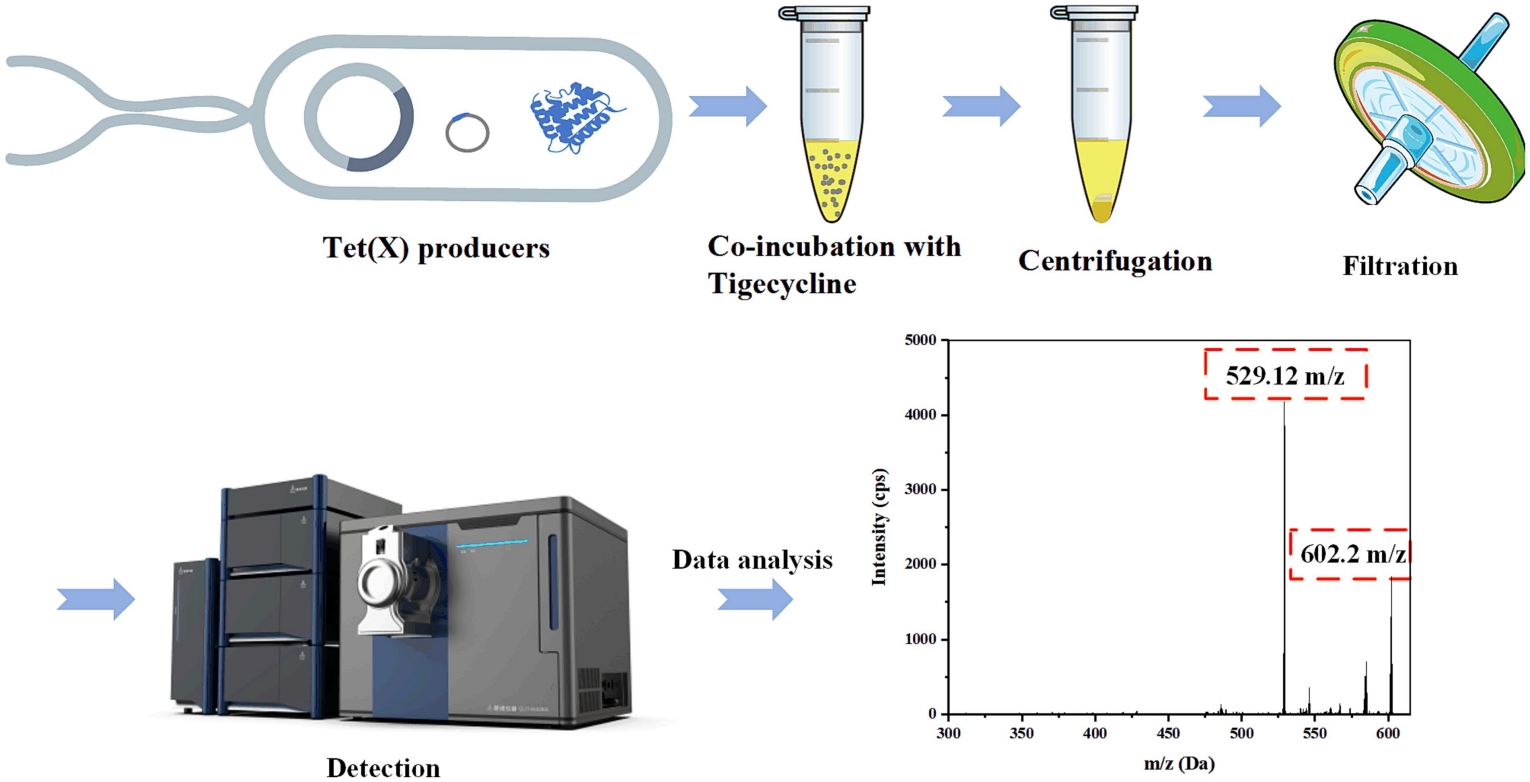
The process of detecting Tet(X)-producers by LC-MS/MS.

Tet(X) protein inactivates tigecycline by covalently modifying at the C11a position of the tetracycline molecule, leading to tigecycline deactivation and subsequent oxygen atom addition. We chose tigecycline as the substrate because tet(X3) and tet(X4) genes contribute to tigecycline, while tet(X1) does not mediate tigecycline resistance (Moore et al., 2005). The bacterial solution (1 μL) was inoculated in 1 mL of Luria-Bertani (LB) medium and reached the logarithmic growth stage at 37°C with 200 rpm. Bacteria were then diluted 1:1,000 times into 10 mL fresh sterile Tripsine Soy Broth (TSB) medium, and grown to OD_600_ = 0.6 at 37°C at 200 rpm. Then the bacteria were centrifuged (3,000 g, 15 min) and the supernatant was discarded. Bacterial deposits were washed three times by PBS and then resuspended in PBS (1 mL). Tigecycline (1 μL) was introduced into the tube resuspended in PBS, and the sample was incubated at a temperature of 37°C and stirred at 200 rpm for the specified duration, then centrifuged again at 3,000 g for 2 min and resuspended in PBS. The above was repeated twice to remove antimicrobial agents that did not enter the cell. After discarding the supernatant, acetonitrile (1 mL) was mixed for 5 min, the solution was centrifuged at 10,000 g for 15 min. Ultimately, the liquid above was filtered using a 0.22 μm organic filter membrane in preparation for analysis. Identification of all experimental strains was facilitated post-treatment utilizing analytical instruments, with a comprehensive procedural outline detailed in Figure 2.

### 2.3 Instrument and analytical conditions

#### 2.3.1 QLIT-6610MD

Sample analysis was performed using the superior quadrupole-linear ion trap tandem mass spectrometry, which was co-developed by the National Institute of Metrology China and a corporate entity (QLIT-6610MD, Registration No. 20222220215, Anhui Limu Medical Device Co., Ltd., Anhui, China). The solution prepared according to the sample pretreatment steps was injected into an Elite C18 column (4.6 mm × 150 mm, 5 μm). Ammonium formate supplemented with 0.2% formic acid and acetonitrile containing 0.1% formic acid were used for mobile phases A and B. The liquid phase method was equivalent, and A:B = 70:30. We used the positive-ion mode. The 0.1 μg/mL solution was injected into the syringe pump and then manually adjusted the MS voltage and all parameters to the optimal value. 4,500 V and 280°C were the voltage and temperature of the ion source, respectively. Sprayer gas (GS1) was pumped at 46 psi, while heater gas (GS2) was pumped at 20 psi. Analyses of QLIT-6610MD were conducted in MRM mode.

#### 2.3.2 AB QTRAP 6500+

At the same time, sample analysis was performed with a Prominence UFLCXR LC system (Shimadzu, MD, United States) coupled to an AB Triple Quad^™^ 6500+ MS/MS (AB Sciex, MA, United States) detector. The liquid phase conditions (the column and mobile phase) are consistent with the QLIT-6610MD. The 0.1 μg/mL solution was injected into the syringe pump, and the MS voltage and all parameters were manually adjusted to their optimal values. During the testing, the turbo-V source was kept at 400°C and the IonSpray voltage was set to 5,500 V. A pressure of 45 psi was set for the nebulizer gas, and 30 psi was set for the heater gas. The ions of tigecycline and the oxidized tigecycline were monitored. The corresponding settings are shown in Table 3. This experiment was carried out at 0.6 mL/min, with a column temperature of 40°C.

**Table 3:** Mass spectromic parameters.

Compound	Parent ion (m/z)	Product ion (m/z)	QLIT-6610MD			AB 6500+		
			Enrichment time/ms	*q* point	Fragmentation energy	Decluster potential/V	Collision energy /eV	Dwell time /ms
Tigecycline	586.1	569.1	150	0.3	25	97	20	100
Oxygenated tigecycline	602.2	529.1	150	0.3	25	97	20	100

### 2.4 PCR amplifications and sequencing

The development of a universal test PCR assay was carried out to detect variations of tet(X). In addition, tet(X3) and tet(X4) were also designed (Tuckman et al., 2007).

### 2.5 Statistical analysis

Receiver operating characteristic curve (ROC) analysis was utilized to identify the cut-off value. The calculation of this parameter is closely related to the Youden index. The ROC curve is a graph that represents the relationship between sensitivity and specificity. The threshold is then established by calculating the Youden index, also known as the correct index, which is a method to evaluate the authenticity of a screening test. The formula for calculating the Youden index is the sum of sensitivity and specificity minus 1. A higher index indicates better effectiveness and greater authenticity of the screening test. By using the ROC curve to find the predictive value at which the Youden index is maximized, the corresponding numerical value is determined as the threshold. The metabolic ratio of tigecycline was calculated as the metabolite/(metabolites + tigecycline), and the results of 50 strains were calculated to establish a threshold ratio distinguishing strains containing the resistance gene tet(X) from those without it. Strains were classified as containing the resistance gene tet(X) if their ratio was better than or equivalent to the cutoff value. Three independent experiments were used to calculate the ratios.

## 3 Results

### 3.1 Identification of tigecycline & oxygenated tigecycline

**Figure 3:**
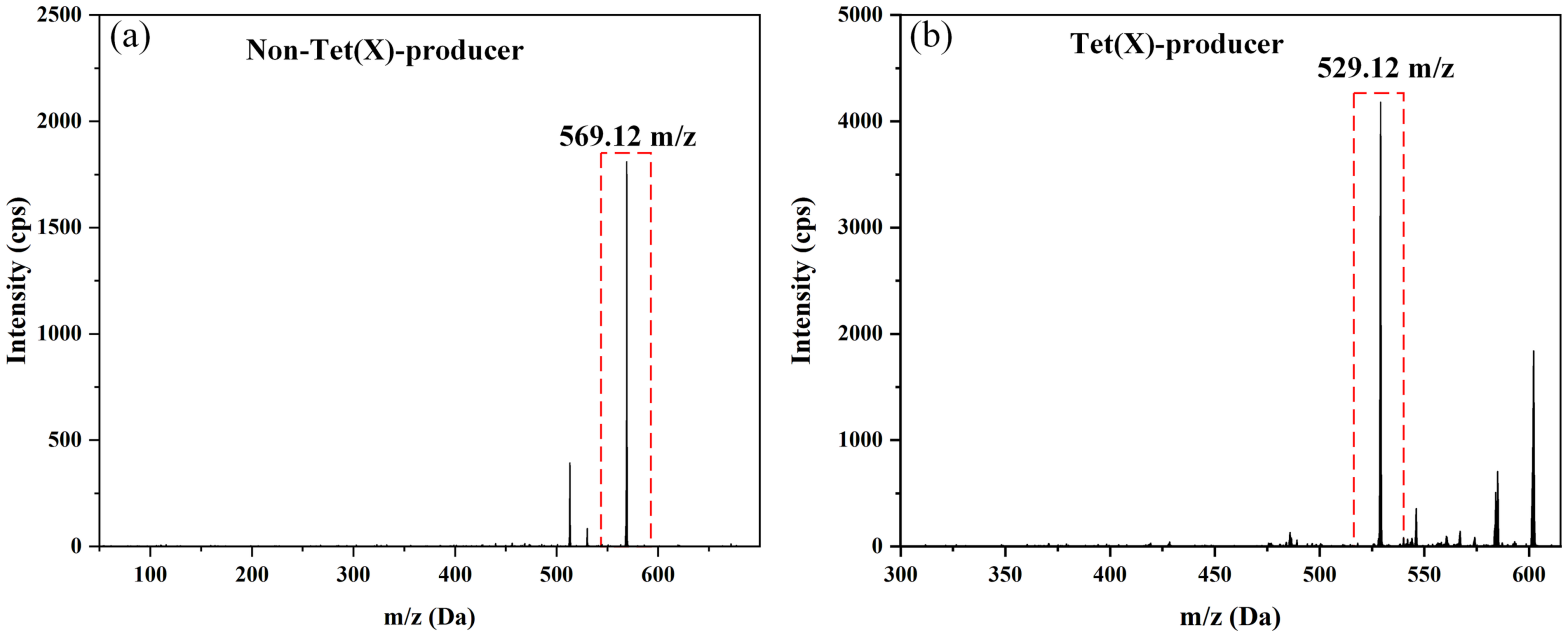
Mass spectra of Non-Tet(X)-producer and Tet(X)-producer. **(A)** The mass spectra of Non-Tet(X)-producer. **(B)** The mass spectra of Tet(X)-producer.

Tigecycline inactivation by tet(X) occurs through a mechanism involving covalent modification at C11a of the tetracycline nucleus, leading to oxy addition (Moore et al., 2005). In this study, the ideal parameters for tigecycline and its oxidative products were initially determined on QLIT-6610MD. The characterized quantitative ion pairing of tigecycline in the ESI positive mode was observed at 586.1 → 569.1 m/z (Figure 3A). The oxidation product exhibited a peak m/z value of 602 ± 0.2, indicative of the addition of an oxygen atom (586 ± 0.2 m/z) to tigecycline (Moore et al., 2005). Although the purified oxygenated tigecycline had yet to be isolated, incubation of tigecycline and bacteria harboring the tet(X) resistance gene at 37°C resulted in the production of its oxidized form. This solution was utilized to refine the MS parameters for oxygenated tigecycline. The predominant compound detected within the solution was the oxidized (+16 Da) form of tigecycline, without observing any other noteworthy reaction by-products. While articles pertaining to the quantitative ion pairs of oxygenated tigecycline have not been documented, analysis of the product ions of oxygenated tigecycline using QLIT-6610MD consistently displayed a loss of 73 Da. Consequently, the finalized SRM for oxygenated tigecycline detection was established at 602.2 → 529.1 m/z. The anticipated appearance of a 529.1 ± 0.2 m/z peak in the mass spectrum of the strain carrying tet(X), as discerned through QLIT-6610MD analysis (Figure 3B), was noted. Conversely, this peak was absent in strain that does not produce tet(X), where instead a 569.1 ± 0.2 m/z peak was observed in their mass spectra.

### 3.2 Development of the LC method

**Figure 4:**
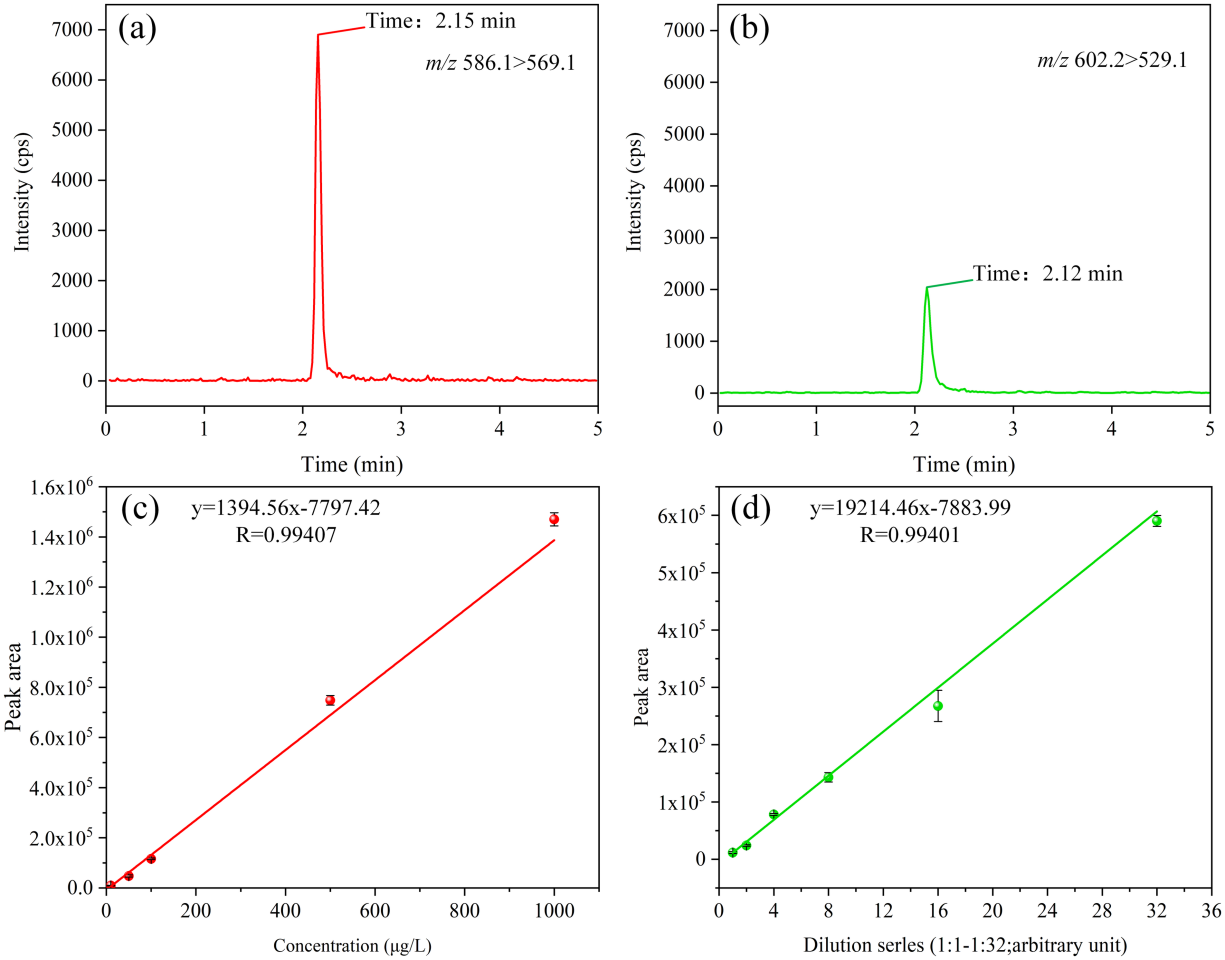
LC parameters for tigecycline and oxygenated tigecycline. **(A)** The retention time of tigecycline. **(B)** The retention time of oxidized tigecycline. **(C)** The linear range of tigecycline. **(D)** The linear range of oxidized tigecycline.

In alignment with the analytical protocol delineated in section 2.3, optimal retention profiles for tigecycline and its oxidation product, oxygenated tigecycline, were attained using an Elite C18 column (4.6 mm × 150 mm, 5 μm). The mobile phase consisted of 10 mmol/L of aqueous amine formate plus 0.2% formic acid (A) and 0.1% acetonitrile formate (B), resulting in retention times of 2.15 min and 2.12 min for the respective compounds (Figures 4A,B). Utilizing QLIT-6610MD, our analysis demonstrated robust sensitivity and linearity, with tigecycline exhibiting a linear range from 10 to 1,000 μg/L (Figure 4C). The method exhibited a limit of detection of 5 μg/L and a limit of quantification of 10 μg/L. Given the non-commercial availability of oxygenated tigecycline, we performed serial dilutions to establish its linearity, revealing excellent linearity even at a 32-fold dilution (Figure 4D). Furthermore, a reliable liquid-phase method was implemented using AB6500+, with detection and quantification limits of 1 μg/L and 2 μg/L for tigecycline, respectively.

### 3.3 Optimization of the analysis protocol

**Figure 5:**
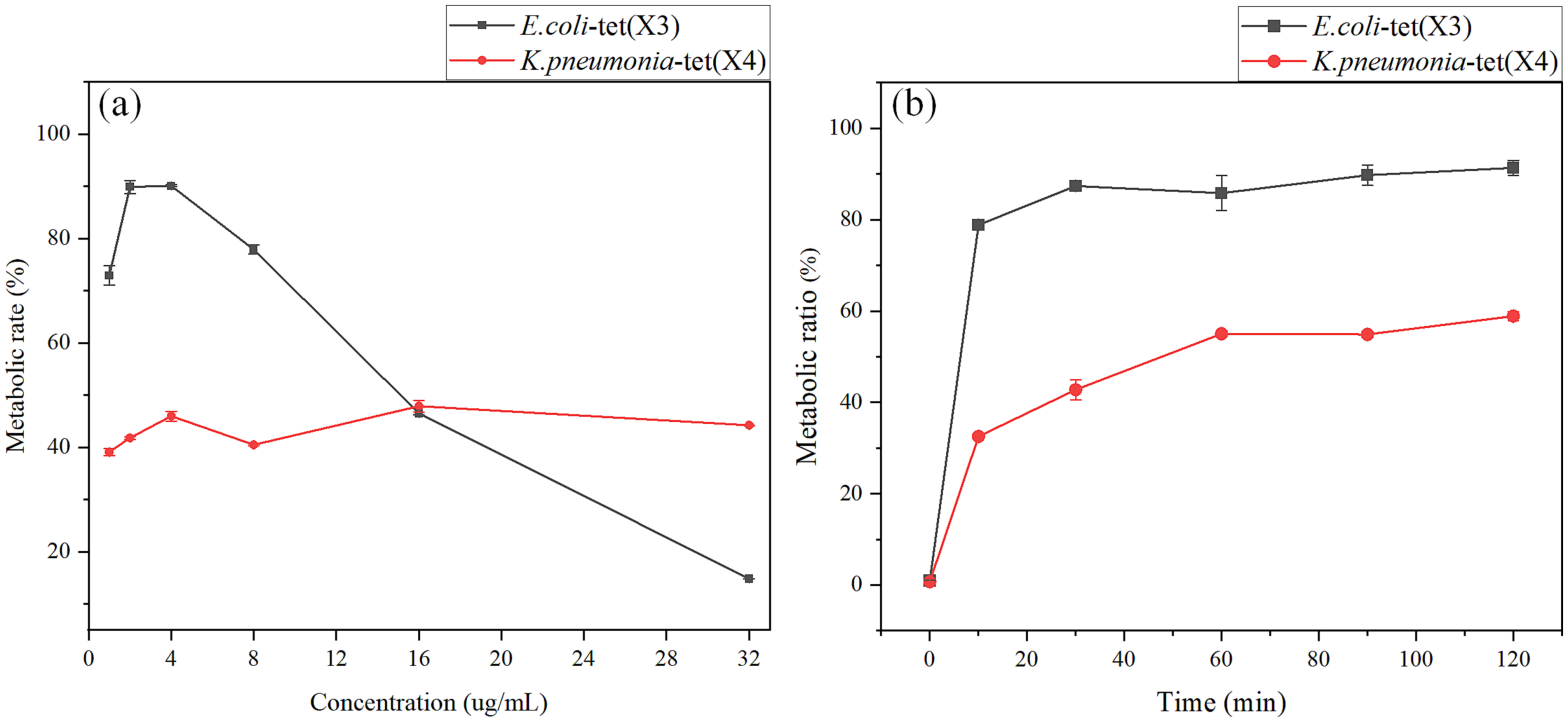
Optimization of tigecycline concentration and the bacterial incubation time. **(A)** Optimization of tigecycline concentration. **(B)** Optimization of the bacterial incubation time.

To devise a rapid and robust liquid methodology for detecting tet(X) in environmental samples, the conditions of the incubation process were meticulously optimized, including the drug concentration and duration. Initially, the study focused on refining the drug addition concentrations during the experimental phase, where varying amounts (1, 2, 4, 8, 16, 32 mg/mL) of tigecycline (1 μL) were added to 1 mL of bacterial suspension. The outcomes are depicted in Figure 5. Notably, at a tetracycline concentration of 4 μg/mL, *E. coli* harboring the resistant gene tet(X3) exhibited the highest metabolic ratio at 90.13%. With escalating drug concentrations in the bacterial fluid, the metabolic ratio demonstrated a declining trend. Akin to *E. coli*, *K. pneumoniae* bearing the resistance gene tet(X4) displayed a substantial metabolic ratio of 45.99% at 4 μg/mL concentration. Although the metabolic ratio fluctuated with increasing drug concentrations in the case of *K. pneumoniae*, the deviation from the preceding metabolic ratio was marginal. Consequently, the optimal drug incubation concentration for this investigation was established as 4 μg/mL in Figure 5A.

In this study, we optimized the time of bacterial incubation during the test process, we mixed the bacterial solution after the addition of tigecycline and incubated in a 37°C shaker at 200 rpm for 0, 10, 30, 60, 90 and 120 min, and the results were shown in Figure 5B. The metabolic ratio of *E. coli* carrying the resistance gene tet(X3) was relatively high at 30 min, which was 87.39%, and there was no significant fluctuation in the metabolic ratio over time. The metabolic ratio of *K. pneumonia* carrying the resistance gene tet(X4) was 42.76% at 30 min of incubation time, and the metabolic ratio reached a relatively stable state at 60 min, 55.02%. Combining the results of the two strains, the optimal incubation time for this study was 60 min. The reason was that this time point provided for stable and rapid detection results.

### 3.4 Creation of the model and calculation of the cut-off value

**Figure 6:**
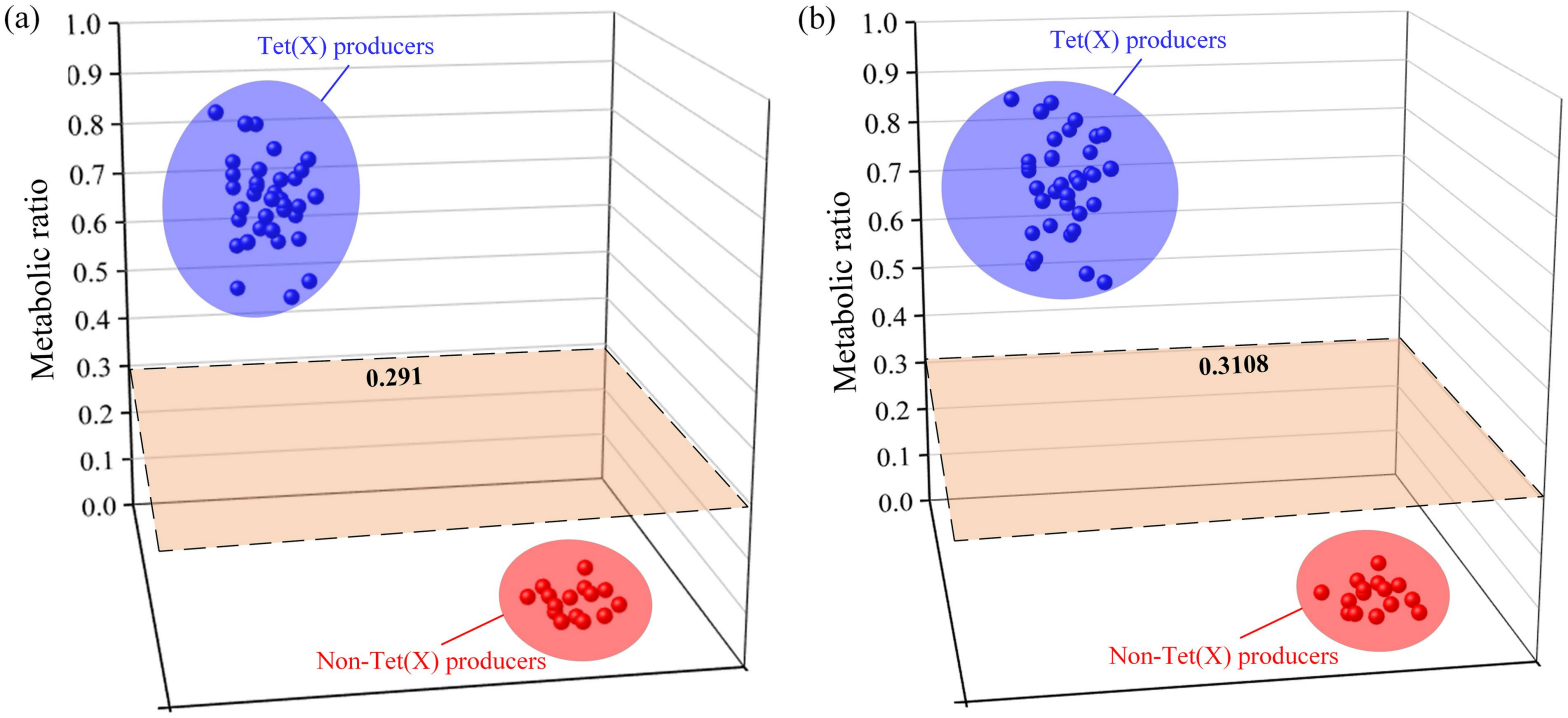
Distribution of liquid-mass combination (LC-MS) metabolic ratios used to establish the cutoff value. **(A)** The cutoff value of QLIT-6610MD. **(B)** The cutoff value of AB 6500+.

Results obtained from 50 strains were utilized for model construction and cut-off value determination. Strains and drug incubation were analyzed using the QLIT-6610MD and AB6500+ instruments following pretreatment. The metabolic proportions calculation effectively distinguished between strains who produce tet(X) and those who do not. The study results were presented in Table 1. For tet(X3)-positive *E. coli*, the analysis with QLIT-6610 indicated a range of 0.726 to 0.874, and for tet(X4)-positive *E. coli*, a range of 0.521 to 0.879. In contrast, *E. coli* lacking the tet(X) resistance gene exhibited ranges from 0.002 to 0.032. Additionally, the seven tet(X4)-positive *K. pneumoniae* strains displayed values ranging from 0.568 to 0.781, while detection in *K. pneumoniae* without the tet(X) gene yielded a value of 0.029. The metabolic ratio range for tet(X)-producing strains was 0.521 to 0.879, contrasting with non-tet(X)-producing strains within the range of 0.002 to 0.032. Employing receiver operating characteristics (ROC) analysis, a metabolic ratio threshold of 0.291 was established to differentiate between strains who produce tet(X) and those who do not, as illustrated in Figure 6A. Strains exceeding the 0.291 threshold were considered as tet(X) producers, while strains below were identified as lacking the tet(X) resistance gene.

Similarly, 4 tet(X3)-positive *E. coli* varied from 0.737 to 0.896, and there were 26 tet(X4)-positive *E. coli* strains with metabolic proportions ranging from 0.564 to 0.899 after AB6500+ analysis of the bacterial solution. The range of *E. coli* testing without the tet(X) resistance gene was 0.016 to 0.061. In addition, the seven tet(X4)-positive *K. pneumoniae* strains ranged from 0.560 to 0.815. The *K. pneumoniae* test without the tet(X) resistance gene was 0.033. The metabolic ratio of strains carrying tet(X) was from 0.560 to 0.899, whereas that of strains that do not produce tet(X) was 0.016 to 0.061. The ROC analysis enabled us to establish a metabolic ratio threshold of 0.3108 to differentiate between strains that produce tet(X) and those that do not (Figure 6B). A strain with a metabolic ratio higher than 0.3108 was categorized as a tet(X) producer, while a strain with a metabolic ratio lower than 0.3108 was identified as not carrying the tet(X) resistance gene.

### 3.5 Model verification

This study rigorously validated a previously established model by examining 70 bacterial strains. These strains underwent blinded testing utilizing a calculated cutoff value, with the detailed outcomes displayed in Table 2. Notably, among the 56 tet(X) producers, we found that 55 out of the 56 strains carrying tet(X) had metabolic ratios that exceeded the designated cutoff level, suggesting the potential utility of this assay in detecting tet(X). The metabolic proportions of strains, as assessed via QLIT-6610MD and AB 6500+ instruments, ranged from 0.418 to 0.908 and 0.453 to 0.911, respectively. Importantly, these metabolic profiles were in complete concordance with the PCR results. Likewise, the 14 non-tet(X) producers scrutinized in the study demonstrated metabolic ratios spanning from 0.016 to 0.059 and 0.011 to 0.051, respectively, mirroring the findings obtained from PCR analysis.

Compared to the traditional PCR technique, the LC-MS/MS tet(X) detection method, when analyzed using the QLIT-6610 and AB6500+ platforms, has shown comparable sensitivity and specificity. Specifically, the QLIT-6610MD group demonstrated a sensitivity of 98.9% and a specificity of 100%, mirroring the performance observed with the AB 6500+ group. These results underscore the high accuracy and reliability of the LC-MS-MS tet(X) method across both instrument platforms, thereby highlighting their comparable detection capabilities.

## 4 Discussion

Tet(X) represents a flavin-dependent monooxygenase identified within *Bacteroides fragilis*, accountable for catalyzing the degradation of tigecycline (Speer and Salyers, 1988). In a significant development in 2017, researchers unveiled the appearance of tet(X3) and tet(X4), harbored on plasmids, capable of conferring resistance to all tetracyclines, including tigecycline (He et al., 2019). Tigecycline, once deemed the ultimate weapon in the armory against bacterial resistance, has now succumbed to reports of resistance. The prevalence of plasmids carrying tigecycline resistance genes within ubiquitous *Enterobacteriaceae* and *Acinetobacter* species portends a formidable challenge in combating infections caused by recalcitrant strains (Jiang et al., 2022). Consequently, establishing a method to observe tet(X) is of utmost urgency.

Phenotypic approaches are increasingly being used to dedetect drug resistance genes in recent years. Several papers have used MALDI-TOF-MS to observe common drug resistance genes. Zheng et al. (2022) established a fast and simple approach for the detection of tet(X) producers in Gram-negative bacteria. Li et al. (2022) established a method to accurately determining colistin resistance to pathogens, and the distinction between sensitive and resistant strains was very clear. However, the LC-MS/MS is considered to have more advantages. When detecting drug-resistant genes, it may be necessary to use abnormally high drug concentrations. On the contrary, in the case of LC-MS-MS for detecting drug-resistant genes, the selection of drug concentrations is more in line with conventional practices. Moreover, the sensitivity and specificity of the LC-MS-MS are relatively high. David presented a new LC-MS/MS method for detecting carbapenemase activity in bacterial isolates, highlighting its robustness with a 100% correlation with the modified Hodge test and PCR, both used as functional tests for carbapenemase production (Peaper et al., 2013). A method utilizing LC-MS/MS was devised to assess the ratio of peak areas resulting from the hydrolysis of meropenem, enabling the detection of carbapenemase activity. This method demonstrated a sensitivity of 97.64% and specificity of 100.00% (Li et al., 2022). Similarly, a LC-MS/MS method was established to determine tet(X) in this study, with a sensitivity and specificity of 98.9 and 100%, respectively. LC-MS/MS was employed for the first time to observe tet(X), with its performance in identifying tet(X) compared to PCR assays as the control.

The efficacy of the LC-MS/MS system in detecting tet(X) was assessed across a cohort of 123 strains, comprising 93 tet(X) producers and 30 non-tet(X)-producers. The identification of tet(X) was achieved through the measurement of the metabolic ratio of tigecycline. In theory, the addition of oxygen to tigecycline results in the appearance of a peak at 602 ± 0.2 m/z, indicative of tet(X) production in the test strain (Cui et al., 2020). The peak fragmented to generate a 529.1 m/z on the LC-MS/MS. In comparison to conventional PCR assays, the accuracy of tet(X) detection within this study was consistently high. Notably, among the 93 tet(X)-producing strains examined, 92 exhibited robust 529 ± 0.2 m/z peaks, with only one strain displaying a diminished response, likely attributable to the reduced activity of the tet(X) enzyme. Conversely, the non-tet(X)-producers manifested relatively weaker 529 ± 0.2 m/z peaks compared to their tet(X)-producing counterparts, possibly due to the introduction of a shared impurity peak during strain and drug incubation. Comparable phenomena have been documented by Cui et al. (2020) utilizing MALDI-TOF MS for tet(X) detection. To guarantee the method’s effectiveness, researchers determined a predefined cutoff value. Through meticulous data analysis, a distinct cutoff value was established to discern the presence of the tet(X) within strains in this study. Results obtained from strains not carrying tet(X) indicated that the observation of weaker 529 ± 0.2 m/z peaks did not compromise the integrity of the LC-MS/MS tet(X) testing outcomes.

The LC-MS-MS method demonstrated a remarkable sensitivity of 98.9% and a specificity of 100% for the quick identification of the tet(X) resistance gene. The development of this model necessitated only a small cohort of strains carrying tet(X) and those not carrying tet(X) to optimize incubation time, drug concentration, and the cut-off value. Notably, in comparison to analogous studies, this method featured shorter incubation time and lower drug concentrations, rendering it highly adaptable to various bacterial species and antibiotics. Nonetheless, it is important to acknowledge the limitation of this study, namely the absence of additional variant testing. Presently, five distinct tet(X) resistance genes have been identified across multiple bacterial strains, including tet(X3), (X4), (X5), (X6), and (X7), all exhibiting formidable resistance against tetracyclines (He et al., 2019; Wang et al., 2019; Gasparrini et al., 2020; Sun et al., 2020). Future research endeavors should prioritize the utilization of LC-MS-MS to observe diverse resistance genes. Moreover, the methodology outlined in this study ought to be expanded to encompass the identification of microorganisms within complex matrices such as blood or urine specimens, with further validation of the LC-MS-MS tet(X) detection protocol being imperative.

## 5 Conclusion

This study introduced a LC-MS/MS assay for monitoring the antibiotic resistance gene tet(X) in environmental samples. The method was based on the ROC curve and Youden’s index, identifying bacteria producing tet(X) by comparing the metabolic ratio of oxidative tigecycline with specific threshold values. This newly developed analytical detection method allows for the rapid and effective identification of bacteria producing tet(X). The method was simple and fast, showing outstanding sensitivity and specificity. When applied to the analysis of 70 bacterial strains, the sensitivity of LC-MS/MS detection compared to PCR was 98.9%, with a specificity of 100%. Additionally, this detection method significantly reduced the testing time, and the reagents used in the process were simple, which greatly reduced the testing costs. In summary, this research provided a new direction for the efficient detection of the resistance gene tet(X) and had also been proven to be easily adaptable for routine implementation in clinical microbiology laboratories equipped with LC-MS/MS instrumentation.

## Data Availability

The original contributions presented in the study are included in the article/Supplementary material, further inquiries can be directed to the corresponding authors.
