# Supplementary material for: A rapid liquid chromatography-tandem mass spectrometry based method for the detection of Tet(X) resistance gene in Enterobacteriaceae
Source: Front Microbiol. 2024 Dec 5;15:1477740. doi: 10.3389/fmicb.2024.1477740 (PMC11659754; doi:10.3389/fmicb.2024.1477740)
Supplement: Supplementary file 1 [file Supplementary_file_1.docx]

Supplementary Material

## Supplementary Figures

1. **The PCR Results of the Strains Used for Model Establishment**

**Supplementary Figure 1.** The PCR results of 15 bacterial strains lacking the tet(X) gene. In the electrophoretic profile, lanes M and 20 correspond to molecular markers of distinct sizes. Lanes 1 to 15 depict the PCR outcomes for negative strains, with lane 16 serving as a negative control, while lanes 17 to 19 serve as positive controls.


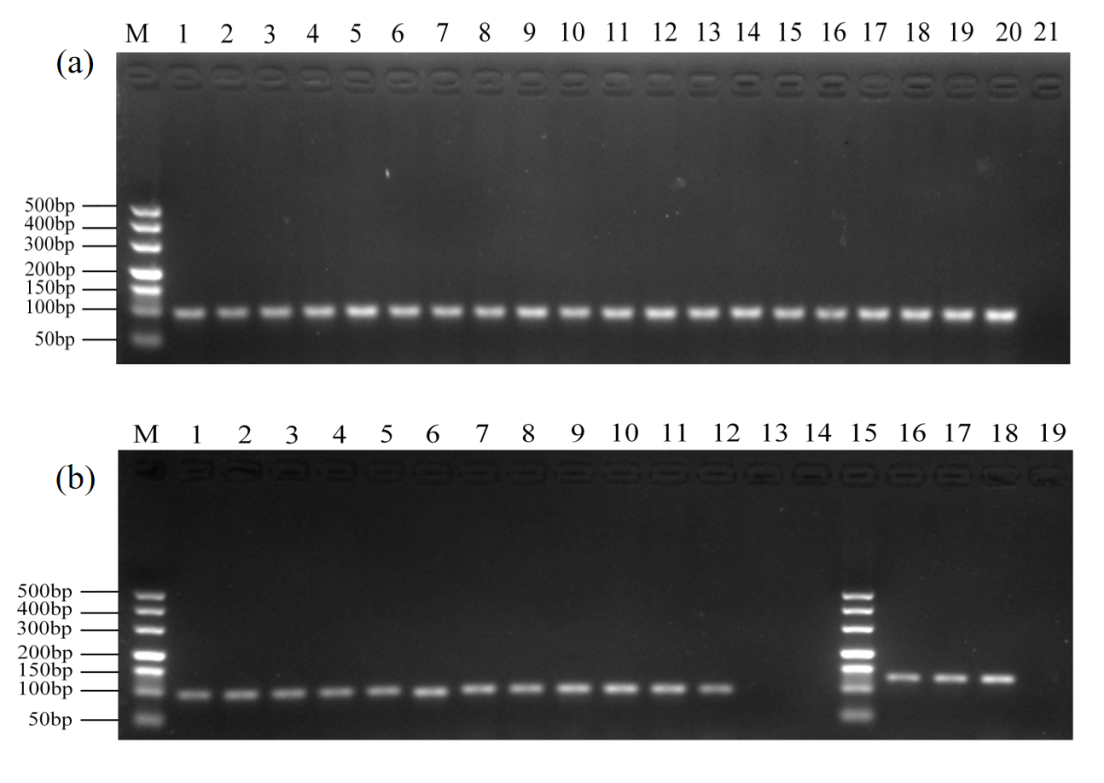


**Supplementary Figure 2.** The PCR results of 35 tet(X)-producers. a. The findings depict the PCR analysis results of 20 bacterial strains harboring the tet(X) gene. Within the electropherogram, lane M is indicative of the molecular marker; Lanes 1 to 20 exhibit the PCR outcomes associated with the tet(X4) gene, with lane 21 allocated for the negative control. b. The PCR analysis outcomes of 15 bacterial strains carrying the tet(X) gene are laid bare. Within the electropherogram, lanes M and 15 signify the molecular markers; Lanes 1 to 12 encapsulate the PCR results linked to the tet(X4) gene, while lane 13 is dedicated to the negative control. Concurrently, lanes 16 to 18 present the results of the tet(X3)-positive strains, contrasting with lane 19 designated for negative control.

1. **The PCR Results of the Strains Used for Model verification**

**Supplementary Figure 3.** The PCR results of 15 bacterial strains lacking the tet(X) gene. In the electrophoretic profile, lanes M and 20 correspond to molecular markers of distinct sizes. Lanes 1 to 9 and lanes 11 to 15 display the PCR results of tet(X) negative bacterial strains, with lane 10 representing the result of the tet(X) positive strain, with lane 16 serving as a negative control, while lanes 17 to 19 serve as positive controls.


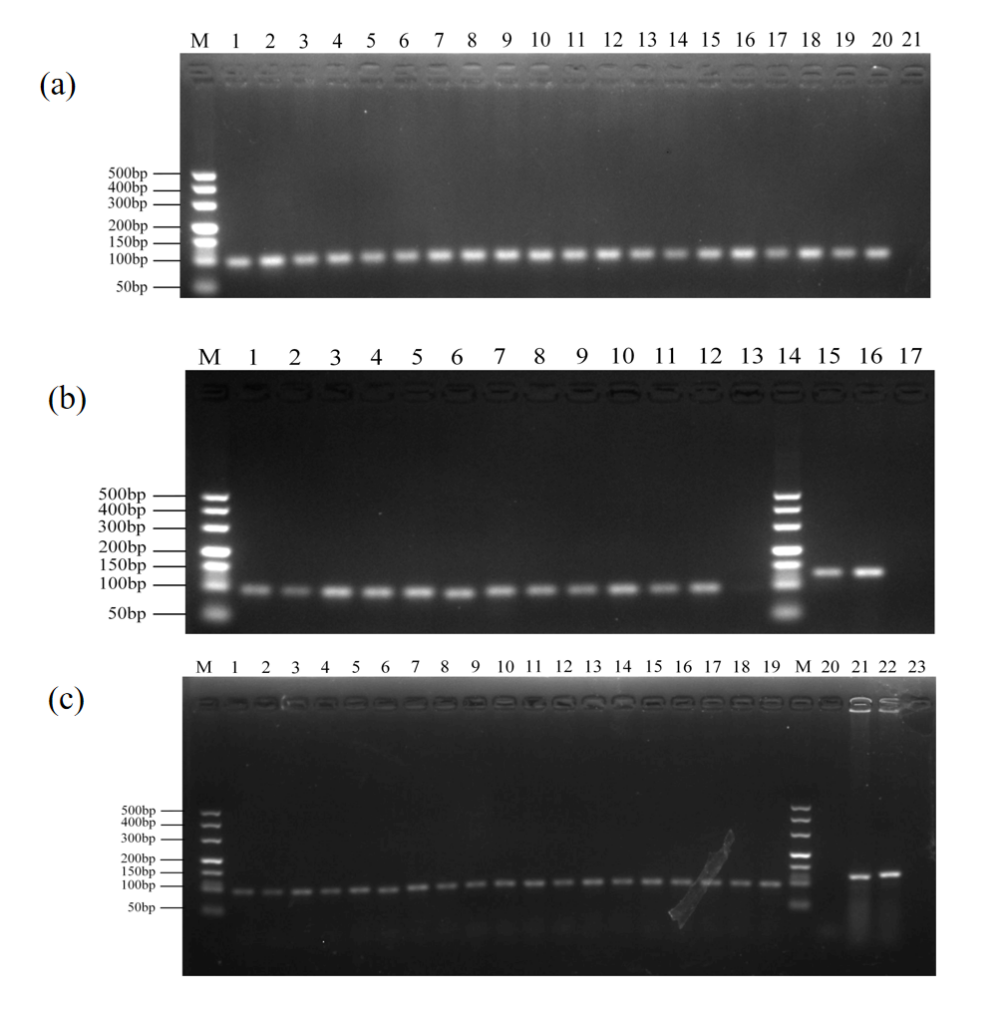


**Supplementary Figure 4. PCR results of 55 tet(X) producing strains**. a. The study outcomes present the PCR analysis results of 20 strains harboring the tet(X) gene. Within the electropherogram, the M lane signifies the molecular marker; Lanes 1 to 20 display the PCR findings linked with the tet(X4) gene, while lane 21 serves as the negative control. b. The PCR results of 14 strains carrying the tet(X) gene are delineated in the electropherogram of Supplementary Figure S4. The M lane serves as the molecular marker; Lanes 1 to 12 exhibit the PCR outcomes associated with the tet(X4) gene, with lane 13 designated for the negative control. Additionally, lanes 15 to 16 represent the findings of the tet(X3)-positive strain, juxtaposed with the negative control found in lane 17. c. The PCR analysis outcomes of 21 strains carrying the tet(X) gene are highlighted in Supplementary Figure S4. Within the electropherogram, the M lane serves as the molecular marker; Lanes 1 to 19 encapsulate the PCR findings associated with the tet(X4) gene, while lane 20 is allocated for the negative control. Furthermore, lanes 21 to 22 display the outcomes of the tet(X3)-positive strains.
